# Deaths in SARS-Cov-2 Positive Patients in Italy: The Influence of Underlying Health Conditions on Lethality

**DOI:** 10.3390/ijerph17124450

**Published:** 2020-06-21

**Authors:** Giovanna Deiana, Antonio Azara, Marco Dettori, Fiorenzo Delogu, Gavino Vargiu, Isabella Gessa, Filippo Stroscio, Marcello Tidore, Giorgio Steri, Paolo Castiglia

**Affiliations:** 1 Department of Medical, Surgical and Experimental Sciences, University of Sassari, 07100 Sassari, Italy; giovanna.deiana90@gmail.com (G.D.); azara@uniss.it (A.A.); castigli@uniss.it (P.C.); 2 Public Health Service, Local Health Unit, 07100 Sassari, Italy; fiorenzo.delogu@atssardegna.it (F.D.); gavino.vargiu@atssardegna.it (G.V.); isabella.gessa@atssardegna.it (I.G.); filippo.stroscio@atssardegna.it (F.S.); 3 Assessorato dell’Igiene e Sanità e dell’Assistenza Sociale, 09123 Regione Autonoma della Sardegna, Italy; mtidore@regione.sardegna.it; 4 Azienda Tutela Salute, 07100 ATS Sardegna, Italy; giorgiocarlodir@libero.it

**Keywords:** SARS-CoV-2, COVID-19, lethality, underlying health conditions, Italy

## Abstract

This study aims to underline the clinical characteristics of patients who died after testing positive for SARS-CoV-2 infection in one region of Italian and to evaluate the influence of underlying health conditions on the fatal outcome. A matched case-control study was designed by analyzing the data regarding positive subjects observed up to April 21, 2020. The case fatality rate was 7.9%, with a higher proportion of deaths in men than women. The specific standardized mortality ratio was 0.15—0.13 for males and 0.2 for females, showing that mortality is much lower than expected. Cardiovascular diseases, chronic lung diseases and diabetes mellitus showed a significant association with the outcome. Although the case fatality rate in Sardinia in regard to age and gender patterns seems to be similar to that for Italy as a whole, its quantitative value was far lower than the national one and possible explanations might include the genetic characteristics of the Sardinian population or the immediate closure of its borders as soon as the epidemic started. Our results highlighted that lethality is strongly dependent on the presence of multiple concomitant serious diseases. It is important to have epidemiological strategies for effective guidance on public health actions in order to improve chances of survival.

## 1. Introduction

Severe acute respiratory syndrome coronavirus 2 (SARS-CoV-2) has been identified as the causative pathogen of an ongoing outbreak of respiratory disease known as coronavirus disease 2019 (COVID-19). The virus spread quickly and the number of people infected is rising dramatically worldwide [1]. The first interpersonal transmission in Italy was reported on February 20, 2020 and now Italy is undergoing one of the largest outbreaks of COVID-19 outside Asia [2].

The fast-evolving nature of the COVID-19 pandemic and the lack of information about this new virus led to exceptional challenges for health care systems as well as to dramatic socio-economic impacts in Europe and worldwide. As of 16 May 2020 a total of 4,425,485 confirmed COVID-19 cases and 302,059 deaths have been reported worldwide [3], while 224,760 confirmed cases and 31,763 deaths have been reported in Italy [4].

Reported diseases ranged from those with few to no symptoms to severe cases where the infection can cause pneumonia, severe acute respiratory syndrome and even death. Various reports indicate advanced age and male gender, which are independent variables, as predictors for severe disease and fatal outcome. Additional risk factors are also reported, including the presence of pre-existing health conditions such as hypertension, type 2 diabetes and chronic obstructive pulmonary disease, which are dependent variables [5,6]. Although it is not clear whether the severity of underlying health conditions affects the risk for severe SARS-CoV-2 infection, the emerging significant differences in lethality are striking.

The case fatality rate (CFR) (number of deaths in persons who tested positive for SARS-CoV-2 divided by the number of SARS-CoV-2 cases) in Italy appears higher than that reported in other European countries and in China (14.1% in Italy, 11.9% in Europe and 5.5% in China on May 16, 2020) [3,4,7] and presents numerous differences in the various regional settings (18.3% in Lombardy in the northern part of Italy and 5.1% in Umbria in the center), highlighting that the geographical spread of the COVID-19 epidemic is very heterogeneous [8]. Nevertheless, the number of deaths cannot be solely attributed to the fact that the epidemic started earlier in Italy compared with other countries.

Despite the publication of national statistics, such information does not allow a complete interpretation of the COVID-19 outbreak in Italy, as the Italian national health service is made up of numerous regional structures which have provided varied regional responses to the emergency, and because of the intrinsic characteristics of the diverse population between and within regions [9].

Sardinia, an island with a population of 1,639,591 inhabitants [10], experienced phenomena such as isolation, endogamy and little genetic flow from the various populations that have invaded the Mediterranean area. This suggests that its population is relatively homogeneous and suitable for epidemiological studies, in particular for the understanding of different pathologies through case-control association studies [11].

The present study focuses on the clinical characteristics of patients dying with SARS-CoV-2 infection in Sardinia and investigates the influence of underlying health conditions on the fatal outcome. Identifying the epidemiological characteristics of the infection might provide evidence for risk stratification and assist in making appropriate decisions to control the outbreak.

## 2. Materials and Methods

### 2.1. Study Population

Sardinia lies in the center of the western Mediterranean Sea and its population has a genetic structure very different from that of the rest of Italy. The genetic drift (variation in gene frequency due to random fluctuations) seems to be the main cause of this difference. Due to its geographical characteristics and in particular to its insularity, Sardinia presents features, such as genetic homogeneity, which make its population suitable for epidemiological studies [12,13].

With regard to the demographic composition, Sardinia is composed of 50.9% females and 49.1% males with several differences within the various age groups. While in the younger age groups the male population is slightly more numerous, large differences are found in the age groups 80−89 and >90 where females account for 60.7% and 70.9% respectively. The population over 65 years of age totals 23.8%, of these 44.2% are male and 55.8% are female [10].

### 2.2. Study Design

A matched case-control study was designed by analyzing the data of the 1223 SARS-CoV-2 positive subjects observed in Sardinia up to April 21, 2020 and whose data were available in the public health department. Information collected regarded age, gender, clinical status (synthetic indicator of the severity of the symptoms), the possible presence of several health conditions and potential risk factors (chronic underlying diseases) and the final outcome.

Of the 97 deceased subjects (45 females and 52 males), underlying health conditions were available for 90 patients (42 females and 48 males). The sample was compared to controls (healed, asymptomatic or pauci-symptomatic subjects) paired (1:1) for age (+/−3 years) and gender, consecutively observed from the 1126 surviving positive subjects at the time of study. The decision to pair cases and controls by age and gender was made in order to reduce the influence of these individual characteristics and focus only on clinical features.

### 2.3. Statistical Analysis

Descriptive statistical analyses were expressed with absolute and relative (percentage) frequencies. CFR was calculated by comparing the number of SARS-CoV-2 deaths with the number of positive SARS-CoV-2 subjects. To assess the differences in mortality for SARS-CoV-2 and eliminate the heterogeneity of the corresponding populations, Sardinian mortality was indirectly standardized with reference to the Italian-specific mortality rate by age group in order to calculate the expected number of deaths [14]. The SARS-CoV-2 standardized mortality ratio (SMR) for the Sardinian population was then obtained by comparing the number of deaths for SARS-CoV-2 observed in Sardinia with the respective number of expected deceased patients. Finally, the 95% confidence interval (95% CI) was calculated as proposed by Vandenbroucke [15].

The conditional logistic regression model was applied to the analysis of matched case-control studies for explanatory variables. The analysis included case status (deceased patients) as the dependent variable and the variables concerning the presence of active tumors (patients being treated), diabetes mellitus, cardiovascular diseases, HIV, chronic lung diseases, chronic renal diseases, other metabolic diseases, obesity, liver diseases, chronic neurological diseases and other pathologies as risk predictors for fatal outcome. Odds ratio (OR) with 95% CI was calculated; the level of significance was established at *p* < 0.05. Statistical analysis was performed using STATA 16.1 (Stata Corp, College Station, TX, US).

## 3. Results

**Figure 1:**
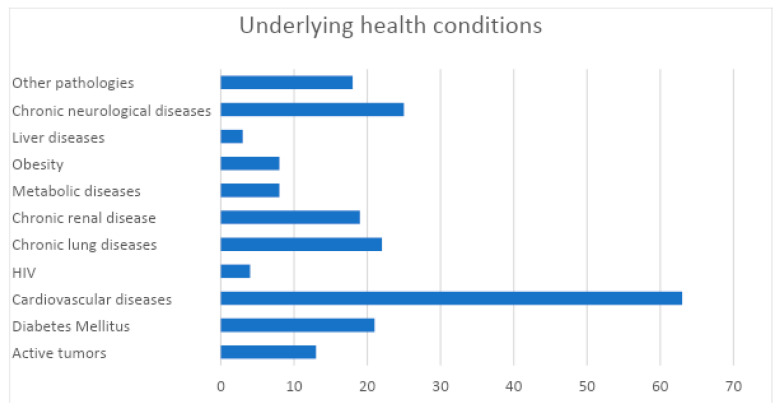
Underlying health conditions of the 90 deceased patients with SARS-CoV-2 in Sardinia.

Among all 1223 SARS-CoV-2 patients observed (724 females and 499 males, 59.2% and 40.8% respectively), 97 deaths occurred (Table 1). Most case patients were in the age group 50−59 (*n* = 238/1223, 19.5%), 5.7% were under the age of 30 and 23% were 80 years of age or older. Among the 97 deaths, 45 were female (46.4%) and 52 male (53.6%). The mean age of patients dying with SARS-CoV-2 infection was 80.4 +/−10.6 (median 82.9, range 42.5−97.5) which is significantly higher (*p* < 0.001) than the mean age of patients who contracted the infection (58.4 +/− 19.9, median 56.1, range 0.1−99.1). Women dying with SARS-CoV-2 were older than men (84.1 +/− 8.9 and 77.1 +/− 11.1 respectively) (*p* < 0.001). Overall, CFR was 7.9%, but cases in those aged 80−89 years had a CFR of 22.9% and cases in those aged 90 years and older had a rate of 18.4%. The number of deaths over 70 years old accounts for 83.5% of the total. No deaths occurred in cases under the age of 40. The analysis clearly shows a higher proportion of deaths with SARS-CoV-2 in men than women, with a lethality percentage of approximately double (10.4% and 6.2%, respectively) (*p* = 0.008). In the age group >90, a higher lethality is observed in females (19% compared to 16.7% of males), albeit not significant, and this may be due to the demographic structure of the population with a very low number of males. No information on underlying health conditions were available for seven deceased patients (7.2%). At least one underlying condition or risk factor was present in 89 out of 90 (98.9%) deceased patients for which data were available. The most commonly reported conditions were cardiovascular diseases (*n* = 63, 64.9%), chronic neurological diseases (*n* = 25, 25.8%), chronic lung diseases (*n* = 22, 22.7%) and diabetes mellitus (*n* = 21, 21.6%) (Figure 1).

Comparing our results with the data provided by the Italian Higher Institute of Health on April 23, 2020, it can be noticed that also in this case, most of the positive cases are in females, albeit with a less marked difference (51.3% females and 48.4% males). Similarly, most cases were in the age group 50–59 (18.4%) and 24.2% were older than 80 years. The Italian CFR, with a crude rate of 13.1%, is much higher than in Sardinia and peaks in the age group 80−89 (30.8%). In this case too, lethality is higher for males than for females (17.1% and 9.3% respectively) (*p* < 0.001) and it was higher in all age groups, unlike what was found in our results [14].

**Figure 2:**
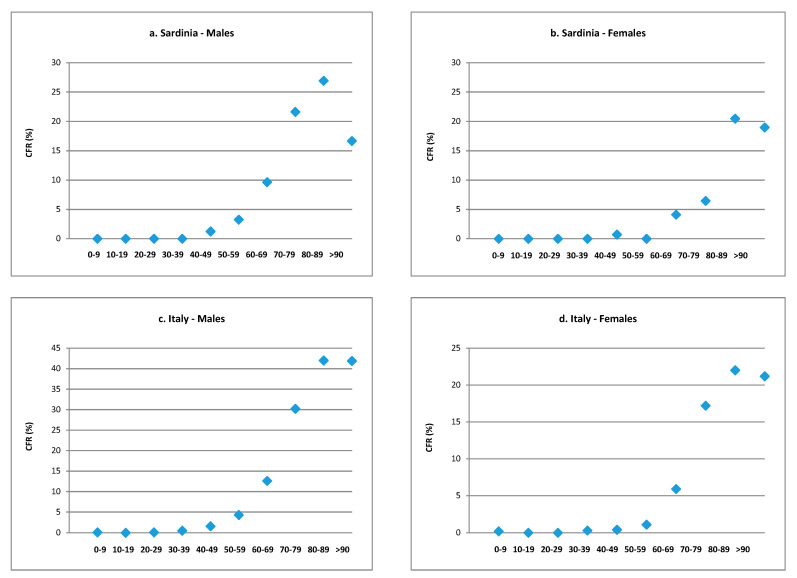
Trajectories of specific SARS-CoV-2 case fatality rate for Sardinian and Italian population by age groups and gender. (**a**) Sardinia—males. (**b**) Sardinia—females. (**c**) Italy—males. (**d**) Italy—females.

The age- and gender-specific trajectories of CFR for both the Sardinian and the Italian population are reported in Figure 2. Apart from the quantitative values, higher in the national population (in particular in males), the shapes of the curves appear similar between the two populations, with a low mortality rate up to the age of 40−49 for men and up to the age of 50−59 for women, followed by steady growth up to the age of 80−89 and a plateau for people over 90, except for a slight fall for men in Sardinia.

Specific SMR was 0.15 (95% CI: 0.12−0.19), 0.13 (95% CI: 0.1−0.17) for males and 0.2 (95% CI: 0.15−0.26) for females, showing that Sardinian specific mortality for SARS-Cov-2 is far lower than expected in respect to Italy.

**Figure 3:**
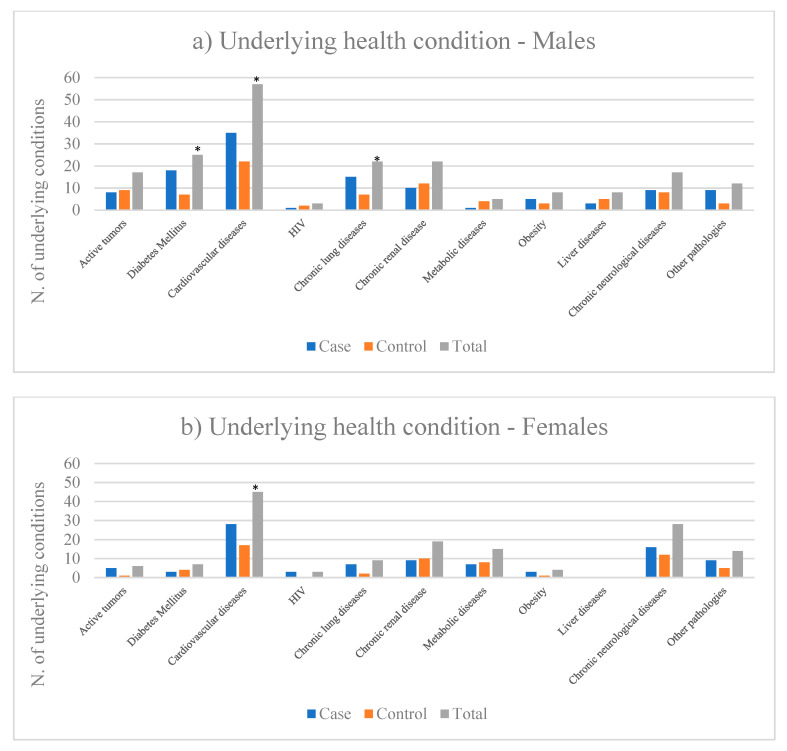
Case control study: underlying health conditions in both male **(a)** and female **(b)** cases deceased with SASRS-Cov-2 and paired controls. * *p*-value < 0.05.

In regard to risk factors, medical records were available for 93% (90/97) of deceased patients. The average number of pathologies observed in this population is 2.3 (median 2, range 0−6). Overall, 3 patients (3.3%) had 0 pathologies, 23 (25.6%) had 1 pathology, 29 (32.2%) had 2 pathologies and 35 (38.9%) had 3 or more pathologies. In Figure 3 the distribution of the observed pathologies for cases and controls is reported by gender. In women, the average number of pathologies observed is 2.1 (median 2, range 0−5), and in men, the average number of pathologies observed is 2.4 (median 2, range 0−6). Both for males and females, statistically significant differences between cases and controls were observed in the prevalence of cardiovascular diseases (*p* <0.05). Statistically significant differences between cases and controls were also observed among males in relation to diabetes mellitus and chronic lung diseases. It is worth noting that among females, while a higher, albeit not significant, prevalence of chronic lung diseases was also observed in cases, a very low prevalence without a significant difference between cases and controls was observed for diabetes mellitus.

Since significant differences in CFR were observed between genders and age groups, we chose to implement a conditional logistic analysis pairing death cases with 1:1 controls for these two variables.

Table 2 shows the results of the conditional regression analysis for the association between case status with available underlying health conditions and the background explanatory variables. The presence of diabetes mellitus (OR = 3.2, *p* = 0.028, 95% CI: 1.1−9.1), cardiovascular diseases (OR = 4.0, *p* = 0.002, 95% CI: 1.7−9.7) and chronic lung diseases (OR = 3.5, *p* = 0.021, 95% CI: 1.2−9.9) showed significant association with the case status. No significant association with case status was found with active tumors, HIV, chronic renal diseases, other metabolic diseases, obesity, liver diseases, chronic neurological diseases and other pathologies (*p* >0.05).

## 4. Discussion

In the present study, we described the clinical characteristics of patients who died while positive for SARS-CoV-2 infection in Sardinia and investigated the influence of underlying health conditions as prognostic factors for fatal outcomes. The report has shown that, in regard to the individual characteristics age and gender, the CFR pattern in Sardinia is substantially similar, at least from a qualitative point of view, to the national one, with some exceptions related to lethality in women of older age. In general, the higher proportion of male deaths can be explained by the natural longer life expectancy of women. Therefore, what remains to be explained is the substantial difference in lethality, from a quantitative point of view. In fact, CFR in Sardinia is far lower than the national one (7.9% and 13.1%, respectively), despite the fact that the island is known for the extreme longevity of the resident population and even boasts many centenarians (*n* = 412) [10]. A possible explanation might be the quality of life or a particular diet. If we consider [16,17], one may wonder if this difference could be related to specific genetic factors, whose variability have progressively decreased due to low immigration rates and facilitated the emergence of specific genetic characteristics that either do not predispose towards or might actually protect from diseases that are major causes of mortality, particularly in the elderly.

Another possible reason could be related to the fact that Sardinia immediately closed all borders as cases arose in Italy and, being an island, it was quite straightforward to shut down all airports and maritime access points, thus avoiding any type of external interference. From that moment on, any person arriving on the island, including those who arrived in the previous 14 days, had to carry out a 2-week isolation period in their own home, further reducing the possibility of contagion. As a consequence, the low number of infections, with most of the municipalities being free of cases, may have protected those population groups at higher risk. In this regard, it is important to consider that there was a delay of about 10 days between the first case in Italy and the first case in Sardinia.

As uncovered by the logistic regression, several concomitant serious diseases were found to be strong predictors for fatal outcome, such as diabetes mellitus, cardiovascular diseases and chronic lung diseases. It is important to consider these factors for risk stratification, as close monitoring and appropriate treatment might help to improve the outcome.

Sardinia, as well as Italy as a whole, has a large proportion of patients with high rates of chronic obstructive pulmonary diseases and ischemic heart diseases, and this might explain, as suggested by Boccia et al. [18], the higher probability of fatal outcome compared to that observed in China. In fact, in patients with cardiovascular diseases and chronic lung diseases, we found a risk of death respectively four and three and a half times higher. In regard to diabetes mellitus, the present study showed that patients affected had a three times higher probability of fatal outcome, although this should be attributed mainly to males. Considering that the Sardinian population has one of the highest incidences in the world of type 1 and type 2 diabetes mellitus [19,20] we would have expected to observe a higher lethality in Sardinia.

Generally speaking, it is important to emphasize that, in Italy, CFR statistics consider COVID-19 related deaths those occurring in patients positive for SARS-CoV-2, independent of pre-existing health conditions, leading to an overestimation of the lethality rate due to the impossibility of differentiating between deaths with SARS-CoV-2 infection and deaths caused by SARS-CoV-2 infection. As a matter of fact, SARS-CoV-2 probably accelerated death in individuals suffering from serious diseases. Moreover, in accordance with central government directives, tests were carried out primarily on symptomatic subjects resulting in a higher proportion of positive tests and an apparently increased CFR [21]. In particular in Sardinia, the number of tests daily performed per 100,000 inhabitants is lower than the Italian average (53 and 88 tests, respectively), almost a half compared to Lombardy and a quarter compared to Triveneto (Trento-Bolzano, Friuli Venezia Giula, Veneto) [22].

At the same time, it can be assumed that some cases have not been recorded for several reasons. First of all, especially in the initial period of the emergency, in some cases the test for SARS-CoV-2 was not performed. Furthermore, some deaths in untested people have been related to organ dysfunctions (heart or kidney) without considering that the virus could have triggered the disorder [23].

This report, like almost all the studies carried out in this period of emergency, presents indeed an important limitation: data collected are preliminary and in a continuous consolidation phase, consequently, results are likely to change as additional data become available. A possible limitation, experienced in different countries, might be the difficulties in accounting for deaths in retirement homes. However, Italy presents a lower proportion of elderly persons living in retirement homes and, in our experience, there have been no difficulties in finding these data. Another further limitation is that the study focused on the influence of underlying health conditions. Nevertheless, the analysis of gender- and age-related lethality could be the subject of further studies. On the other hand, the studies in this first phase of pandemic are fundamental in order to fully understand the role of comorbidity in the risk of death. In fact, recent acquisitions of knowledge in the therapeutic field, such as the efficacy of the use of hydroxychloroquine, azithromycin, low molecular weight heparin, etc., tend to significantly alter the risk of death with SARS-Cov2 infection [24,25]. Moreover, several further strengths may be identified. Firstly, the peculiar characteristics of Sardinian history made its population optimal for case-control studies. Second, all data collected have a good reliability, coming from the public health department. Furthermore, the statistical analysis of our study was strong in its consistency.

## 5. Conclusions

In summary, this study was planned to underline the clinical characteristics of patients dying with SARS-CoV-2 infection and to evaluate the influence of underlying health conditions on the fatal outcome. To our best knowledge, this is one of the first reports analyzing lethality in SARS-CoV-2 positive patients taking into consideration an entire Italian Region and evaluating independent and dependent variables separately, calculating to what extent clinical features affect lethality.

As our results highlighted that lethality is strongly dependent on the presence of multiple concomitant serious diseases, it is essential to adopt epidemiological strategies to ensure adequate protection to these high-risk groups and to offer effective guidance on public health actions in order to improve chances of survival. It is worth highlighting the importance of further investigating which characteristics might have decreased lethality in Sardinia compared to the rest of Italy.

Additionally, given the little experience in dealing with the new virus, it is compulsory to maintain a high level of attention and to continue monitoring the evolution of the phenomenon. Indeed, health authorities at the national, regional and global levels should increase constant surveillance to be prepared for any evolution of the pandemic and to avoid strategic mistakes.

## Figures and Tables

**Table 1:** Distribution of diagnosed cases and reported deaths with SARS-CoV-2 in Sardinia by age group and gender in respect to lethality in Italy.

Gender		Age Groups											
		0–9	10–19	20–29	30–39	40–49	50–59	60–69	70–79	80–89	>90	ND	All
**Males**	No. of cases	4	10	14	46	80	92	83	74	78	18	0	499
	% of cases	36.4	52.6	35.9	38.3	36.2	38.7	53.2	54.4	38.0	23.7	0	40.8
	No. of deaths	0	0	0	0	1	3	8	16	21	3	0	52
	% of deaths	0	0	0	0	50	100	72.7	80	44.7	21.4	0	53.6
	Lethality %	0	0	0	0	1.3	3.3	9.6	21.6	26.9	16.7	0	10.4
**Females**	No. of cases	7	9	25	74	141	146	73	62	127	58	2	724
	% of cases	63.6	47.4	64.1	61.7	63.8	61.3	46.8	45.6	62.0	76.3	100	59.2
	No. of deaths	0	0	0	0	1	0	3	4	26	11	0	45
	% of deaths	0	0	0	0	50	0	27.3	20	55.3	78.6	0	46.4
	Lethality %	0	0	0	0	0.7	0	4.1	6.5	20.5	19.0	0	6.2
**Total**	No. of cases	11	19	39	120	221	238	156	136	205	76	2	1223
	%	0.9	1.6	3.2	9.8	18.1	19.5	12.8	11.1	16.8	6.2	0.2	100
	No. of deaths	0	0	0	0	2	3	11	20	47	14	0	97
	%	0	0	0	0	2.1	3.1	11.3	20.6	48.5	14.4	0	100
	Lethality %	0	0	0	0	0.9	1.3	7.1	14.7	22.9	18.4	0	7.9
**Italy**	Lethality %	0.2	0	0.1	0.4	0.9	2.6	10.0	24.9	30.8	26.1	0	13.1

**Table 2:** Conditional regression analysis for the association between case status (patients deceased with SASRS-Cov-2 and paired control patients) and underlying health conditions.

Outcome	Odds Ratio	*p*-Value	95% CI
Active Tumors	1.3	0.643	0.4–3.8
Diabetes Mellitus	3.2	0.028	1.1–9.1
Cardiovascular Diseases	4.0	0.002	1.7–9.7
HIV	1.8	0.582	0.2–13.5
Chronic Lung Diseases	3.5	0.021	1.2–9.9
Chronic Renal Disease	0.9	0.888	0.4–2.3
Metabolic Diseases	0.4	0.146	0.1–1.3
Obesity	1.1	0.908	0.4–2.9
Liver Diseases	0.4	0.291	0.1–2.4
Chronic Neurological Diseases	1.3	0.582	0.5–2.9
Other Pathologies	2.1	0.185	0.7–6.3
